# Assessment of the household availability of oral rehydration salt in rural Botswana

**DOI:** 10.11604/pamj.2013.15.130.2793

**Published:** 2013-08-10

**Authors:** Swetha Bindu Jammalamadugu, Botsang Mosime, Tiny Masupe, Dereje Habte

**Affiliations:** 1 University of Botswana-School of Medicine, Botswana

**Keywords:** Oral, Rehydration, Salt, ORS, availability, Botswana

## Abstract

**Introduction:**

Diarrhea contributed for 17.6% of under-five deaths in Botswana. Oral rehydration salt (ORS) therapy has been the cornerstone in the control of morbidity and mortality secondary to diarrheal diseases. The study was aimed at assessing the household availability of ORS following the nationwide campaign of availing ORS at household level.

**Methods:**

A cross sectional community based study was conducted in August 2012. EPI random walk method was used to identify households. Data was collected using interviewers' administered structured questionnaire. SPSS software was used in data entry and analysis.

**Results:**

Oral Rehydration Salt (ORS) was available in 50.8% of the households with under-five children. Information on ORS is well disseminated whereas only three-fourth of informed participants had adequate knowledge of ORS preparation. The sources of information were predominantly the Child Welfare Clinic (88.8%). Being grandmother as a care taker was a negative predictor of household availability of ORS (AOR 0.25, 95% CI 0.09-0.69) while respondents who are knowledgeable about ORS preparation were more likely to have ORS available at home (AOR 1.92, 95% CI 1.10-3.34).

**Conclusion:**

The campaign has brought a significant coverage in terms of availability of ORS. The health education and community sensitization efforts need to go beyond health facilities via other means like the media and community based approaches. Approaches aimed at improving the knowledge of care takers on the importance of ORS, its preparation, correct use and restocking are of paramount importance. Availing community based outlet for ORS is an alternative to enhance accessibility.

## Introduction

Globally there were 6.9 million deaths of children under the age of five years in 2011. The causes in two-third of the deaths were preventable infectious diseases. The largest global burden of childhood death is found in Sub-Saharan Africa housing 23 countries with Under-five mortality rate above 100 deaths per 1000 live births. The 2011 under-five mortality rate in Botswana was reported to be 26 deaths per 1000 live births [1]. A systematic analysis in 2008 estimated that the contribution of diarrheal diseases towards childhood mortality was 15% globally and 19% in Africa alone [2]. In 2007, Diarrhea and Pneumonia were reported as the major causes of under-five deaths in Botswana (17.6% and 14.7% respectively)[3].

Oral Rehydration Salt (ORS) therapy is one of the recognized evidence based interventions used to reduce child morbidity and mortality in diarrheal diseases worldwide. Other interventions include breast feeding, complementary feeding, water, sanitation, hygiene, Rotavirus vaccine, Antibiotics, Zinc and Vitamin-A [4–13]. Availability of ORS at community level has been shown to reduce diarrhea related mortality among under-five children [4]. Combining ORS with other interventions has also been shown to significantly increase the extent of reduction in diarrhea related morbidity [14].

Oral rehydration therapy has been the cornerstone in the control of morbidity and mortality secondary to diarrhoeal diseases since 1979. With the increased use of ORS around the world, the overall mortality secondary to diarrhoeal disease has dropped dramatically [5]. In April 2012 the Ministry of Health of Botswana launched a national campaign to ensure that all households with children under the age of 5 years have ORS and Zinc tablets readily available at home. This approach was suggested in response to the increasing number of children presenting severely dehydrated to the health facilities with resultant deaths. The aim of this study was therefore to assess the household availability of ORS following the introduction of the nationwide campaign.

## Methods

### Study site and population

A descriptive cross-sectional study was conducted in Kweneng District of Botswana. According to the 2011 housing and population census, Kweneng district has an estimated total population of 304,674 with a density of 9.8 persons per square kilometer [15]. The study participants were recruited from Molepolole village which is the headquarters of Kweneng district. This village has a population of 67,598 [15]. The study was conducted amongst households in Molepolole with children under the age of 5 years. The participants of this study were parents or guardians of children under the age of 5 years.

### Sample size and sampling

The sample size was determined using EpiInfo 3.5.3 software. The coverage of the current ORS availability was assumed to be 25%. There is no estimate for household availability of ORS and one quarter of the households with under-five children were expected to have ORS at the early phase of the nationwide campaign. With margin of error of 5% and 5% level of significance, a sample size of 288 was targeted. In the actual data collection, a sample size of 295 was attained. EPI-random walk method was used to select the households around the areas served by the 4 clinics in Molepolole.

### Data collection and analysis

A questionnaire was adapted from the standard Demographic and Health Survey questionnaire [16]. The questionnaire was prepared and administered using the local language. Five enumerators trained on the data collection instrument collected the information. The principal investigators were involved in the supervision of the data collection. Data was collected between August 11-13, 2012.

Epi-Info software version 3.5.3 was used for data entry and descriptive data analysis while SPSS software version 20 was used for bi-variate and multi-variate analysis. Frequency, percentage and mean were computed to describe the findings. The crude and adjusted odds ratio (COR/ AOR) and 95% Confidence Interval (CI) were analyzed to explore associations and a p-value of 0.05 or less was considered statistically significant.

### Ethical issues

Ethical approval was obtained from the University of Botswana Institutional Review Board and Botswana Ministry of Health Research unit. Permission was granted by the local administrators and written informed consent was secured from each participant before the conduct of the interview.

## Results

A total of 295 care takers of children were enrolled in the study with mean age of 35 and a standard deviation of 13 years. The study participants were predominantly females with the large majority reporting some form of formal education. Mothers and grandmothers constituted one-third and one-six of the sample population respectively (Table 1).

**Table 1:** Background characteristics of study participations, Molepolole, 2012

Characteristic	Frequency	Percentage
**Age**		
16-30	144	48.8%
31-40	69	23.4%
41 and above	81	27.5
No response	1	0.3%
**Gender of parent/guardian**		
Male	10	3.4%
Female	285	96.6%
**Education Level**		
No education	32	10.8%
Primary	69	23.4%
Junior Secondary	117	39.7%
Senior Secondary	52	17.6%
Tertiary	20	6.8%
No response	5	1.7%
**Relationship to the child**		
Mother	196	66.4%
Father	7	2.4%
Grandmother	48	16.3%
Sister	2	0.7%
Uncle	2	0.7%
Aunt	9	3.1%
Other relatives	24	8.1%
Non-relative guardian	7	2.4%

Over 98% of the participants reported possession of information regarding Oral Rehydration Salt. However the proportion of the participants who have adequate knowledge regarding the preparation and use of ORS were 219 (74.2%). Their source of information was predominantly from the Child Welfare Clinic (88.8%). Other sources of information reported included hospital (3.8%), friends (4.7%), Radio (4.1%), Television (3.1%), Clinic Card (1.8%) and school (1%). Printed materials like Pamphlets, Posters or Books were mentioned as sources of information by 1.7% while electronic media and social mobilization were not mentioned by any one. The large majority (99%) of the study participants reported that their child attended Child Welfare Clinic regularly.

The household availability of ORS and Zinc Sulphate tablets were 50.8% and 30.2% respectively. Of 145 households without ORS at home, 62 (42.7%) reported to have used up the available ORS while 56 (38.6%) never had it at home. Of 206 households without Zinc Sulphate at home, 120 (58.3%) never had access to the tablet and 54 (26.2%) reported to have used up their supply (Table 2).

**Table 2:** Availability of ORS and Zinc Sulphate at households, Molepolole, 2012

Characteristic	Frequency	Percentage
**Availability of ORS at home**		
Yes	150	50.8%
No	145	49.2%
**Reason for unavailability of ORS**		
Child doesn't have diarrhoea	19	13.1%
Expired packets	1	0.7%
Never given	56	38.6%
Used up	62	42.7%
Unsure	4	2.8%
No response	3	2.1%
**Availability of Zinc Sulphate at home**		
Yes	89	30.2%
No	206	69.8%
**Reason for unavailability of Zinc Sulphate**		
Child doesn't have diarrhoea	15	7.3%
Never given	120	58.3%
Used up	54	26.2%
Unsure	8	3.9%
Thrown away	1	0.5%
No response	8	3.9%

**Figure 1:**
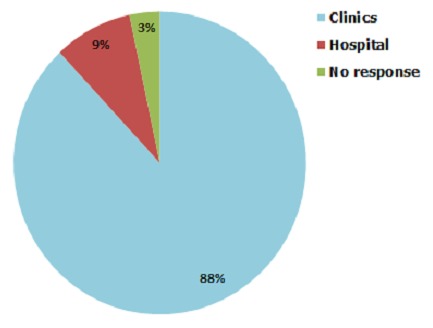
Source of medication among households keeping ORS at home

The sources of ORS among the 150 households with ORS were the Child welfare Clinic (88%) and a public hospital (8.7%) while 3.3% did not specify the source (Figure 1). Of the 89 households with Zinc Sulphate tablets readily available, they obtained it from Child welfare clinic (89.9%), public hospital (7.9%) and private pharmacy (1.1%).

Being grandmother as a care taker was a negative predictor of household availability of ORS (AOR 0.25, 95% CI 0.09-0.69) while respondents who are knowledgeable about ORS preparation were more likely to have ORS available at home (AOR 1.92, 95% CI 1.10-3.34) (Table 3).

**Table 3:** Factors associated with availability of ORS at home, Molepolole, 2012

Characteristic	ORS available at home		Crude OR (95% CI)	Adjusted OR (95% CI)
	Yes	No		
**Age**				
16-30	77	67	1.00	1.00
31-40	33	36	0.79 (0.45, 1.42)	0.69 (0.37, 1.28)
41 and above	40	41	0.85 (0.49, 1.46)	1.64 (0.62, 4.39)
**Relationship of care taker**				
Parent	111	92	1.00	1.00
Grandmother	17	31	**0.46 (0.24, 0.87)**	**0.25 (0.09, 0.69)**
Other care takers	22	22	0.83 (0.43, 1.59)	0.68 (0.33, 1.41)
**Education Level**				
No formal education	19	13	1.00	1.00
Primary	31	38	0.56 (0.24, 1.31)	0.46 (0.19, 1.13)
Secondary and above	96	93	0.71 (0.33, 1.51)	0.53 (0.22, 1.33)
**Knows how to prepare ORS**				
Yes	120	99	**1.86 (1.09, 3.16)**	**1.92 (1.10, 3.34)**
No	30	46	1.00	1.00

## Discussion

Reducing diarrhea morbidity and mortality isa key to achieving the fourth MillenniumDevelopment Goal of reducing child mortality by two-thirds by 2015 (MDG4) [17]. ORS therapy has been shown to play significant role in the reduction of child mortality secondary to diarrheal disease [4, 5, 14]. The findings from this study highlight the areas that need attention in order to improve knowledge, availability and use of ORS.

It is noted that while information on ORS is widely available, only three-fourth of informed participants had adequate knowledge of ORS preparation. Adequate knowledge refers to acquaintance to the method of reconstitution from the packet and daily preparation. A similar finding was reported by a study conducted in rural Bangladesh in 2002 where two-third of the respondents who recognized ORS packet knew how to prepare ORS correctly [18]. It is an indication that the health education efforts need to go beyond information dissemination. More effective measures are needed to empower caretakers on the preparation and use of ORS.

Health facilities especially the Child Welfare Clinics were the principal sources of information about ORS and Zinc Sulphate. The large majority of the children are regular attendees of Child Welfare Clinics and using such an important opportunity to propagate child health information including correct use of ORS is regarded as crucial to extending knowledge and improving practice and use of ORS. The media, printed material and community mobilization were mentioned as sources of information by the minority. One tenth of the care takers never had a formal education and health education approaches need to take in to account such segments of the population. The health information at the Clinics needs to be augmented by other approaches including community based intervention to maintain knowledge and enhance behavior change. Intervention studies have demonstrated that audio-visual and community based approaches were effective in improving knowledge about ORS, child health and survival [19, 20].

Studies done in Botswana to assess the practice of keeping ORS and or Zinc sulphate tablets at home prior to the April 2012 national campaign could not be found in the literature. It is however postulated that keeping ORS and/or Zinc Sulphate at household level used to be uncommon practice before the launching of the campaign. The level of availability of ORS and Zinc Sulphate few months after the campaign is encouraging. The availability of ORS was better than that of Zinc Sulphate. This may be explained by Zinc Sulphate tablets being less readily available in the health facilities or health workers not dispensing the tablets as required.

Some caretakers without ORS or Zinc Sulphate were not replenishing the medications once used up. The health educators need to emphasize on the need to replenish the medications once used up. Accessibility of health facilities might also be a hindrance in the re-fill and an alternative way of community outlets for the medications might be helpful as was demonstrated by a study conducted in Bangladesh [4]. A good number of the participants reported never having had access to either ORS or Zinc Sulphate. The opportunity at the Child Welfare Clinic needs to be exploited such that the clinic can distribute and replenish ORS/ Zinc Sulphate at the follow-up visits to all attendants. Care takers who already own the medications can also be used as change agents in mobilizing other households with ORS unavailable at home.

The study demonstrated that grandmother caretakers were less likely to have ORS at home. This calls for targeted health education efforts to increase knowledge and bring about behavior change among the grandmother caretakers. Participants who are knowledgeable about ORS preparation and correct use were more likely to have ORS available at home. Knowledgeable parents or guardians will make sure that ORS is available at home and use it appropriately. This makes increasing parents and guardian's knowledge about ORS much more imperative and a more effective way of ensuring that most households have a knowledgeable caretaker.

The study finding needs to be interpreted with some caution. The study did not directly assess the association between home availability of ORS and the treatment of children with recent history of diarrhea, and positive outcome of morbidity and mortality reduction. The use of non-probability sampling is also another limitation to note which will affect the generalizability of the findings to populations outside of the study area.

## Conclusion

The Botswana nationwide ORS distribution campaign appears to have contributed to significant population coverage in terms of availability of ORS at household level. The effort at the Child Welfare Clinics alone might not be sufficient to capitalize on the achievement and sustain the improvement in the coverage. The health education and community sensitization efforts need to be intensified through incorporating other means of communication such as the media and community based approaches. Approaches aimed at improving the knowledge of care takers on the importance of ORS, its preparation, correct use and restocking are of paramount importance. Further attention needs to be paid to sensitizing uneducated and older care takers in order to maximize the potential overall benefits of the health promotion messages. Availing a community based outlet for ORS is an alternative option to enhance accessibility. Further study is recommended to assess the ultimate direct benefit of ORS availability in the reduction of morbidity and mortality from childhood diarrheal disease in Botswana.
